# Evaluation of intracellular signalling pathways in response to insulin-like growth factor I in apoptotic-resistant activated human hepatic stellate cells

**DOI:** 10.1186/1755-1536-2-1

**Published:** 2009-01-30

**Authors:** Alessandra Gentilini, Benedetta Lottini, Marco Brogi, Alessandra Caligiuri, Lorenzo Cosmi, Fabio Marra, Massimo Pinzani

**Affiliations:** 1 Dipartimento di Medicina Interna, Università degli Studi di Firenze, Florence, Italy; 2 Center for Research, High Education and Transfer 'DENOThe', Università degli Studi di Firenze, Florence, Italy

## Abstract

**Background:**

Human hepatic stellate cells have been shown to be resistant to apoptotic stimuli. This is likely dependent on the activation of anti-apoptotic pathways upon transition of these cells to myofibroblast-like cells. In particular, previous studies have demonstrated an increased expression of the anti-apoptotic protein Bcl-2 and a decreased expression of the pro-apoptotic protein Bax during the transition of the hepatic stellate cell phenotype from quiescent to myofibroblast-like cells. However, the role and expression of other key anti-apoptotic and survival pathways elicited by polypeptide growth factors involved in the chronic wound healing process remain to be elucidated. In particular, insulin growth factor-I promotes chemotactic and mitogenic effects in activated human hepatic stellate cells and these effects are mediated by the activation of PI 3-K. The role of insulin growth factor-I as a survival factor in human hepatic stellate cells needs to be substantiated. The aim of this study was to evaluate the involvement of other key anti-apoptotic pathways such as PI-3K/Akt/p-Bad in response to insulin growth factor-I.

**Results:**

Insulin growth factor-I induced activation of Akt followed by Bad phosphorylation after 15 minutes of incubation. These effects were PI-3k dependent since selective inhibitors of this molecule, wortmannin and LY294002, inhibited both Akt and Bad phosphorylation. The effect of insulin growth factor-I on the activation of two downstream targets of Akt activation, that is, GSK3 and FHKR, both implicated in the promotion of cell survival was also investigated. Both targets became phosphorylated after 15 minutes of incubation, and these effects were also PI-3K-dependent. Despite the activation of this survival pathway insulin growth factor-I did not have a remarkable biological effect, probably because other insulin growth factor-I-independent survival pathways were already maximally activated in the process of hepatic stellate cell activation. However, after incubation of the cells with a strong apoptotic stimuli such as Fas ligand+cycloheximide, a small percentage of hepatic stellate cells underwent programmed cell death that was partially rescued by insulin growth factor-I.

**Conclusion:**

In addition to Bcl-2, several other anti-apoptotic pathways are responsible for human hepatic stellate cell resistance to apoptosis. These features are relevant for the progression and limited reversibility of liver fibrosis in humans.

## Background

Fibrosis and cirrhosis represent the consequences of a sustained wound healing response to chronic liver disease induced by a variety of causes, including viral, autoimmune, drug-related, cholestatic and metabolic damage. The excessive accumulation of extracellular matrix occurs in most types of chronic liver disease [1-5]. A key role in fibrogenesis has been attributed to hepatic stellate cells (HSCs), which have been identified as major collagen-producing cells in an injured liver.

Following liver injury of any etiology, HSCs undergo a response known as 'activation', which is the transition of quiescent cells into proliferative, fibrogenic and contractile myofibroblasts (HSC/MFs) [1-5].

Numerous studies, performed in animal models of acute or chronic liver injury, have shown a potential reversibility of liver fibrosis and cirrhosis [6]. Recovery from injury in these animals is associated with apoptosis of the HSC/MF and, as a consequence, a reduction in the tissue inhibitor of metalloproteinase (TIMP) levels and progressive degradation of the fibrotic matrix [7-9].

In vitro studies, performed in rat HSCs, have investigated the potential mechanisms regulating HSC apoptosis [10]. Rat HSCs have been shown to undergo apoptosis following treatment with the pentapeptide GRGDS (Gly-Arg-Gly-Asp-Ser), recombinant matrix metalloproteinase 9, an antibody against focal adhesion kinase, Fas/fas ligand, nerve growth factor (NGF), tumour necrosis factor α (TNF-α), interferon gamma, selective peripheral benzodiazepine receptor ligands, and gliotoxin [11,12]. In addition, evidence has been provided concerning possible candidate survival factors preventing HSC apoptosis, including transforming growth factor 1, TIMP-1 and insulin-like growth factor I (IGF-I) [1,10]. Overall, these studies have conveyed the message that HSC apoptosis represents an important limiting step in the fibrogenic process, particularly upon the discontinuation of chronic tissue damage. In addition, these observations have highlighted the possible reversibility of fibrosis and even cirrhosis in humans [1,6].

However, these assumptions are based on animal models where the extent and duration of tissue damage is limited and short-lasting and on studies performed on rat HSCs. Importantly, recent data by Novo et al. [13] suggest that the dynamics of apoptosis in human HSCs could be remarkably different from those observed in rat HSCs. Activated human HSCs were shown to survive with prolonged serum deprivation, exposure to Fas ligand, NGF, TNF-α, doxorubicin, ectoposide, oxidative stress mediators and 4-hydroxynonenal, thus indicating a strong resistance of these cells to programmed cell death. In this connection, these authors showed that the process of HSC activation is accompanied by remarkable changes in the expression of some key proteins involved in the control of apoptosis, and in particular, a shift towards a higher Bcl2/Bax ratio protein expression.

Based on this initial report, the aim of the present study was to further characterise the pathways modulating the apoptotic process in activated human HSCs. In order to maximise this effort, the expression and regulation of different cytoplasmic and nuclear protein systems were evaluated before and following stimulation with IGF-I, a factor known to support growth, metabolism, differentiation and prevention of apoptosis in many cell types [14]. Although IGF-I is produced by many tissues, liver IGF-I synthesis accounts for 90% of the circulating peptide. In particular, liver IGF-I is synthesised at high levels in hepatocytes in response to growth hormone stimulation [15], and in multiple non-parenchymal cell types including HSC [16]. These cells express IGF-I receptor and are important targets for IGF-I action. In cultured HSCs, IGF-I enhances proliferation [17], migration [18] and collagen synthesis [19], providing indirect evidence that IGF-I could play a role in the expansion of activated HSCs and liver fibrosis.

In previous studies [18], we investigated the intracellular pathway of human HSCs involved in both the mitogenic and chemotactic effects. In particular, it was shown that the activation of PI-3K and ERK is required for both IGF-I-dependent HSC proliferation and chemotaxis, confirming an interaction between PI-3K/Akt and MAPK/ERK pathways. The aim of this study was to investigate the intracellular survival signal induced by IGF-I and its possible biological effect.

## Materials and methods

### Materials

Enhanced chemiluminescence (ECL) reagents and nitrocellulose membrane Hybond-C extra were from Amersham Pharmacia Biotech. (Cologno Monzese, Milano, Italy), IMMOBILON Western reagents were from the Millipore Corporation (Billerica, MA, US) IGF-I and platelet-derived growth factor (PDGF) from Peprotech EC Ltd (London, UK), Fas ligand (FasL) from Upstate Biotech. (Lake Placid, New York, US). Antibody against Bad, Akt (n-19) and poly (ADP-ribose) polymerase (PARP) were from Santa Cruz Biotechnology (Santa Cruz, California, US), all other antibodies were from Cell Signaling Technology (Danvers, MA, US). Iscove's medium was from Invitrogen (Carlsbad, CA, US). Annexin-V-FLUOS staining kit was from Roche (Mannhein, Germany). All other reagents were from Sigma Chemical Co. (Sigma Aldrich Spa, Milano, Italy).

### Cell isolation and culture

The use of human material was approved by the Human Research Review Committee of the University of Florence, where cells were isolated and characterised from surgical wedge sections of human livers not suitable for transplantation, as described elsewhere [19]. Cells obtained from samples of different normal human livers were cultured in Iscove's medium supplemented with 20% foetal bovine serum. After reaching confluence in the primary culture, serial passages were obtained, always applying a 1:3 split ratio. Cells were used between serial passages 4 and 7. At this stage of culture, HSCs show phenotypic features of fully activated HSC/MFs and a profile of cell surface markers identical to that of 'interface' MF described in fibrotic and cirrhotic human livers [20,21]. HSC/MFs were plated to obtain the desired subconfluence level (70–80%) and then incubated for 24 hours in serum-free Iscove's medium in order to obtain cells at the lowest level of spontaneous proliferation before the addition of the different stimuli.

### Western blot

Cells were lysed with 50 mM (4-(2-hydroxyethyl)-1-piperazineethanesulphonic acid (HEPES) buffer pH 7.5, 150 mM NaCl, 10% glycerol, 1% Triton X-100, 1.5 mM MgCl_2_, 1 mM ethylene glycol tetraacetic acid (EGTA), 10 μg/ml leupeptin, 10 μg/ml aprotinin, 1 mM phenylmethylsulphonyl fluoride and 100 mM sodium fluoride for 20 minutes at 4°C. Cells were scraped from dishes and centrifuged at 15,000 g for 20 minutes at 4°C. Supernatants were loaded for sodium dodecyl sulphate polyacrylamide gel electrophoresis (SDS-PAGE) gel. After transferring the proteins, blots were incubated with the desired primary antibodies and then incubated with peroxidase conjugated anti-mouse or anti-rabbit immunoglobulins in Tris-buffered saline-Tween containing 1% (weight/volume) non-fat dry milk and developed with ECL reagents or IMMOBILON Western reagents (chemiluminescent-HRP substrate) according to the manufacturer's instructions.

### Akt activity

An immune complex kinase assay of Akt activity was performed as described elsewhere [22]. Briefly, 100 mg of proteins were immunoprecipitated with anti-Akt antibodies followed by adsorption to protein G-agarose. Immunoprecipitates were then collected by a brief centrifugation and washed three times with washing buffer (20 mM HEPES (pH 7.5), 40 mM NaCl, 50 mM NaF, 1 mM ethylenediaminetetraacetic acid (EDTA), 1 mM EGTA, 0.5% Nonidet P-40, 20 mM b-glycerophosphate, 0.5 mM sodium orthovanadate, 1 mM phenylmethylsulphonyl fluoride, 10 mg/ml leupeptin, 10 mg/ml pepstatin and 10 mg/ml aprotinin). The assay was performed by resuspending the beads in kinase buffer (50 mM HEPES (pH 7.5), 100 mM NaCl, 10 mM MgCl_2_, 10 mM MnCl_2_, 10 mM b-glycerophosphate and 0.5 mM sodium orthovanadate) in the presence of 1 mM protein kinase A inhibitor peptide, 50 mM unlabelled ATP and 6 μCi of [γ-32P] ATP, using exogenous histone H2B (1.5 mg/assay tube) as the substrate and incubating for 20 minutes at room temperature. Reaction products were run in a 12% SDS-PAGE, stained with Coomassie Blue and visualised by autoradiography.

### Evaluation of apoptosis

Evaluation of cell apoptosis was performed by evaluation of PARP and caspase cleavage on Western blot.

### Statistical analysis

All Western blots were representative of at least three to four experiments with similar results. Statistical analysis was performed by student's t-test. *P *values = 0.05 or 0.01 were considered significant.

## Results

**Figure 1:**
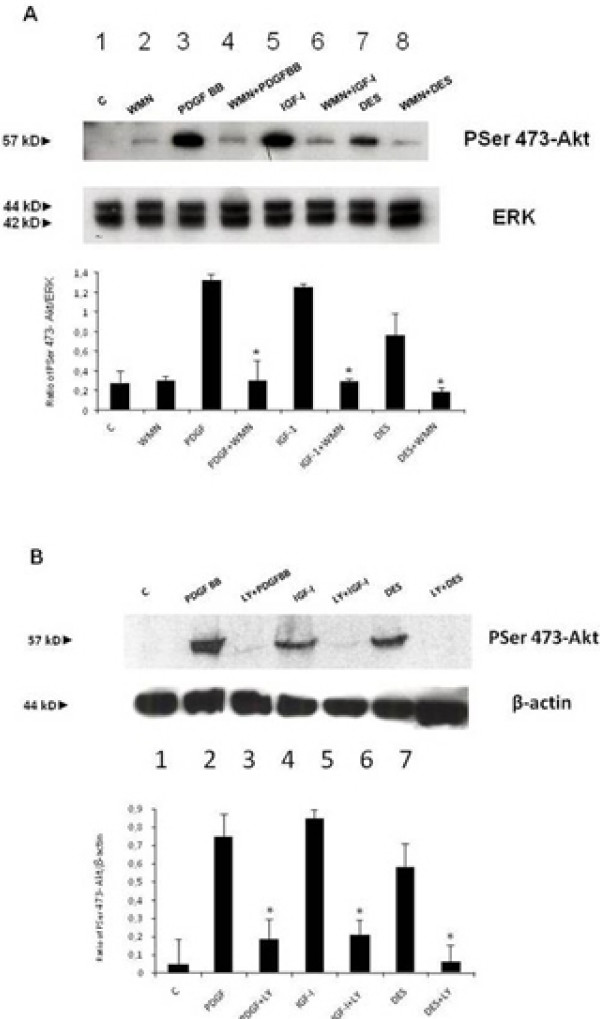
**Hepatic stellate cell extracts (20 μg proteins) were run on 10% sodium dodecyl sulphate polyacrylamide gel electrophoresis gel and analysed by Western blot for pSer 473-Akt**. Fifteen minutes of incubation with 100 ng/ml of insulin-like growth factor I (IGF-I) induced phosphorylation of c-Akt in Ser 473 (lane 5 panel A and lane 4 panel B). This effect was PI-3K dependent since it was blocked by pre-incubation (30 minutes) of hepatic stellate cells with 100 nM WMN (panel A) or 100 μM LY294002 (panel B), two established inhibitors of PI-3K. Platelet-derived growth factor was used as a positive control for p-Akt and DES-(1–3) IGF-I was used as a positive control for IGF-I. Barograms summarise the results obtained in three independent experiments: * *P *< 0.05 or a higher degree of significance when compared with stimulation with growth factors without inhibitor.

**Figure 2:**
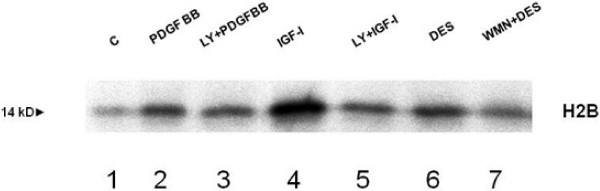
**Activity of Akt measured by labelled phosphorylation of the exogenous histone 2B**. Autoradiography shows that insulin-like growth factor I (IGF-I) induced an increase in Akt activity (lane 4) when compared with the control and this effect was reversed by pre-incubation (30 minutes) with LY294002 (lane 5). DES-(1–3) IGF-I (lane 6) is an analogue of IGF-I that binds to the receptor but does not bind to the IGF-binding proteins.

**Figure 3:**
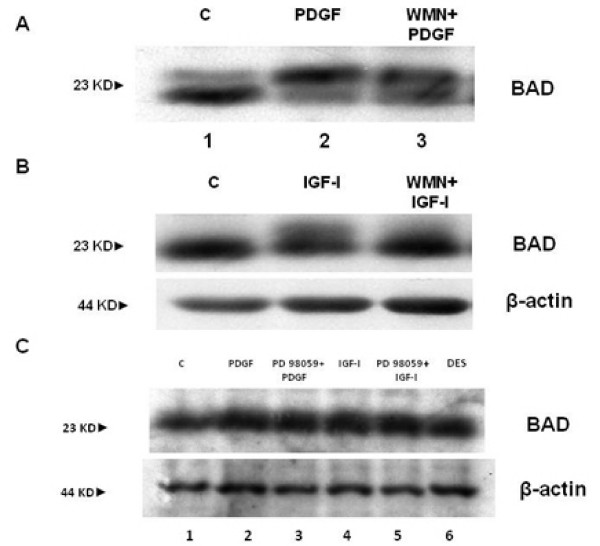
**Proteins (20 μg) obtained from cell extracts were run on a 12% sodium dodecyl sulphate polyacrylamide gel electrophoresis and analysed by Western blot for Bad**. Bad phosphorylation, shown by the protein shift, was evident for hepatic stellate cells treated with platelet-derived growth factor (PDGF) (panel A) and insulin-like growth factor I (IGF-I) (panel B). This effect was PI3-K dependent (lane 3), since it was inhibited by pre-incubation with WMN. Panel C shows the Western blot for Bad obtained with extracts from cells treated with PDGF (lane 2), IGF-I (lane 4) and DES (lane 6) for 15 minutes. It is evident that pre-incubation with PD98059 for 30 minutes did not have any effect on PDGF- and IGF-I-induced phosphorylation (lanes 3 and 5).

**Figure 4:**
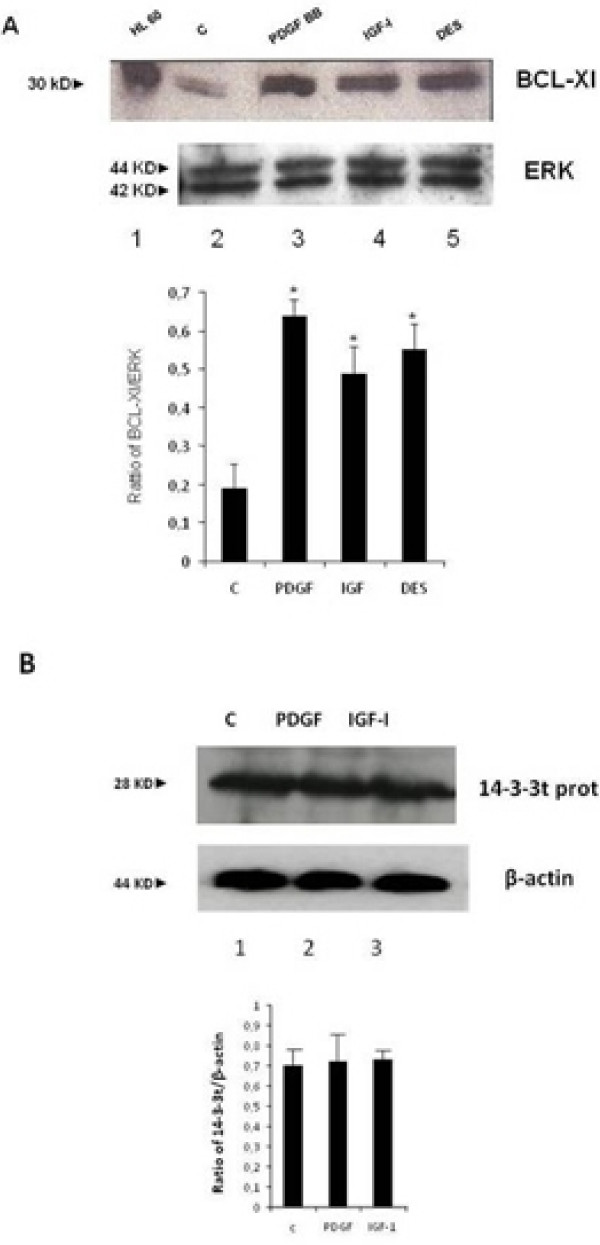
**Delayed effects of insulin-like growth factor I on the expression of proteins involved in the association of Bad**. Insulin-like growth factor I (IGF-I), after 24 hours incubation, induced an increase of expression of the pro-apoptotic protein BCL-Xl (panel A, lane 4) but did not modify the expression of the 14-3-3t protein (panel B, lane 3) that associates with Bad. Barograms summarise the results obtained in three independent experiments: * *P *< 0.05 or a higher degree of significance when compared with the control.

In the first set of experiments we investigated the IGF-I intracellular signalling downstream of PI-3K activation. As shown in Figure 1, IGF-I induced phosphorylation of c-Akt on Ser 473 residue after 15 minutes of incubation. This effect was PI-3K-dependent since it was blocked by pre-incubation of HSCs with 100 nM WMN (Figure 1, panel A) or 100 μM LY294002 (Figure 1, panel B), two inhibitors of PI-3K. DES-(1–3) IGF-I, an analogue of IGF-I able to interact with the IGF-I receptor without the interference of IGF binding proteins, was used as a positive control for IGF-I action. PDGF was used as a positive control for the activation of PI-3K. To confirm that phosphorylation on Ser 473 induced Akt activity [23], an Akt activation assay was then performed. Figure 2 illustrates the activity of c-Akt measured by labelled phosphorylation of the exogenous histone 2B. Autoradiography showed that IGF-I induced an increase in Akt activity when compared with the control and this effect was reversed by pre-incubation with LY294002 or WMN, thus confirming a PI-3k activation dependency. Subsequently, we verified the AKT-induced phosphorylation of Bad, a pro-apoptotic protein, whose pro-apoptotic action is blocked by phosphorylation and consequent association with the 14-3-3t protein [24]. Cells were stimulated with PDGF (Figure 3, panel A) and IGF-I (Figure 3, panel B). Both growth factors were able to induce Bad phosphorylation after 15 minutes of incubation, an effect that resulted at least in part to be PI3-K dependent. Since pre-incubation of cells with WMN or LY294002 could not completely reverse IGF-I-induced Bad phosphorylation, we studied the involvement of ERK in this effect. Pre-incubation of HSCs with PD98059, an inhibitor of ERK activity, did not affect PDGF and IGF-I-induced Bad phosphorylation, thus excluding an involvement of ERK/MAP kinase as a regulatory mechanism (Figure 3, panel C). Protein expression of Bcl-xl and 14-3-3t was then evaluated after 24 hours of incubation with IGF-I and PDGF. As shown in Figure 4, panel A, both growth factors increased Bcl-xl expression, while 14-3-3t protein expression was not modified (Figure 4, panel B). This observation suggests that IGF-I is able to protect cells from apoptosis not only after short-term stimulation but also for as long as 24 hours.

**Figure 5:**
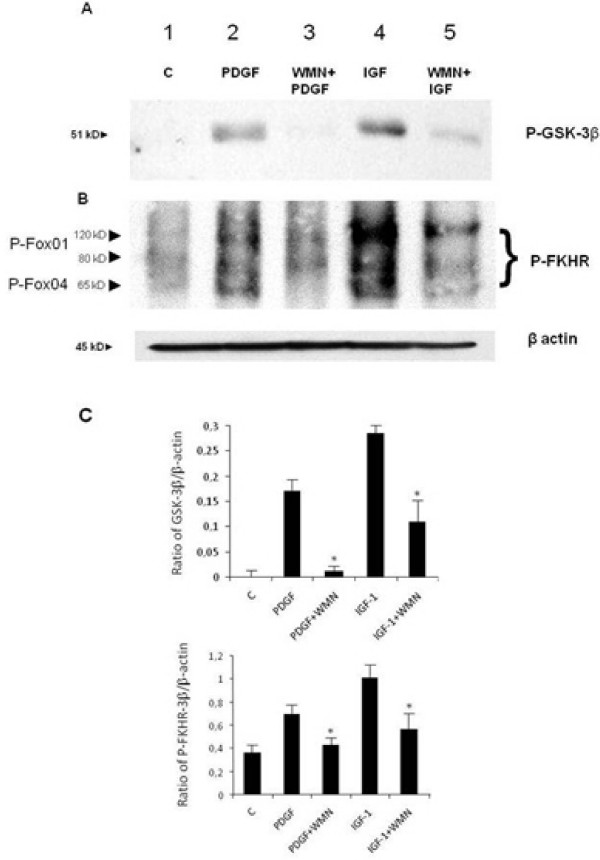
**Effect of insulin-like growth factor I on the phosphorylation of two targets, glycogen synthase kinase 3β (panel A) and FKHR (panel B), downstream of Akt activation**. Both GSK3β and FHKR were phosphorylated after 15 minutes of incubation with insulin-like growth factor I (lane 4) and the action was PI-3K dependent (lane 5). Barograms summarise the results obtained in three independent experiments (Panel C). * *P *< 0.05 or a higher degree of significance when compared with stimulation with growth factors without inhibitor.

The effect of IGF-I on the activation of other proteins downstream of the activation of Akt was also investigated. The best-characterised Akt targets are the Forkhead box O (FOXO) family of transcription factors and glycogen synthase kinase 3β (GSK3β). FOXO proteins (FKHR, FKHR-L1 and AFX) regulate different processes through transcriptional effects on a large number of gene targets [25]. In resting conditions FOXO activates pro-apoptotic factors and cell-cycle-inhibitory proteins, while its Akt-induced phoshorylation leads to a lack of activation of target proteins. GSK3β regulates different cellular processes by phosphorylating many substrates including metabolic enzymes, transcription factors, cell-cycle-regulatory proteins and cytoskeletal proteins. This protein kinase is unusual, as it is generally highly active in resting cells but inhibited in response to cellular signals, in particular through the PI-3K/Akt pathway [26-28]. As shown in Figure 5, phosphorylation of both GSK3β and FKHR was PI-3K dependent after 15 minutes of incubation with IGF-I, confirming an important role of this growth factor in cell-cycle and apoptosis regulation.

**Figure 6:**
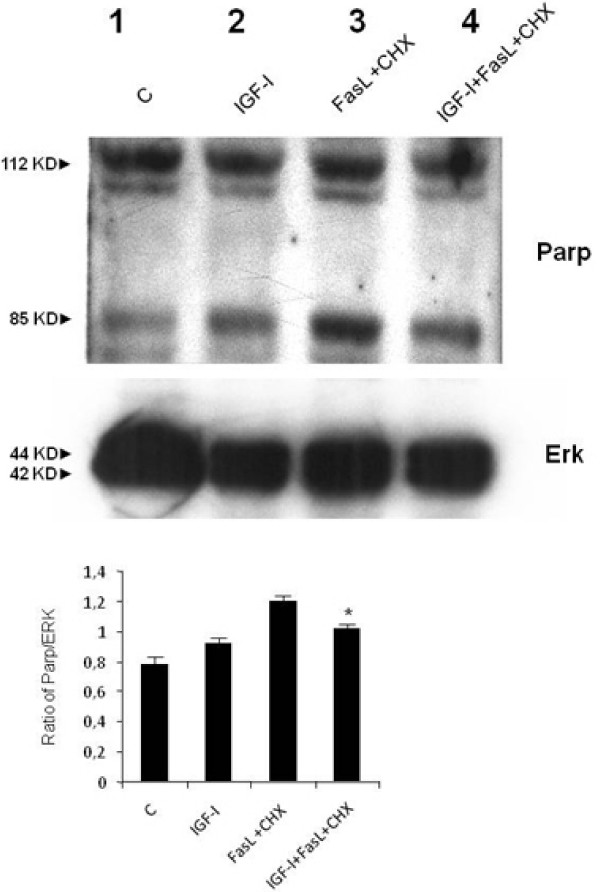
**Western blot analysis for poly (ADP-ribose) polymerase expression**. The analysis was conducted with 20 μg of proteins extracted from hepatic stellate cells treated with insulin-like growth factor I(IGF-I), FasL (20 ng/ml)+CHX (50 μg/ml) or IGF-I+FasL+CHX for 24 hours As shown, FasL+CHX could induce cleavage of poly (ADP-ribose) polymerase (lane 3) and this effect was partially inhibited by IGF-I (lane 4). The barogram summarises the results obtained in two independent experiments: * *P *< 0.05 when compared with incubation with FasL+CHX without IGF-I.

**Figure 7:**
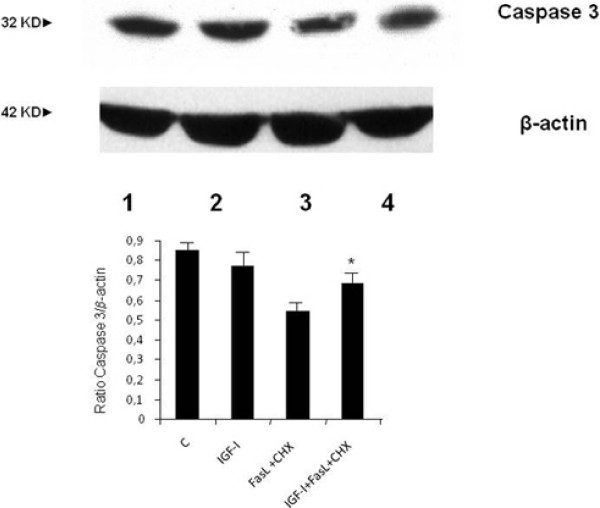
**Western blot analysis for caspase 3**. Hepatic stellate cells were incubated with insulin-like growth factor I (IGF-I) (lane 2), FASL+CHX (lane 3) and IGF-I+Fas+CHX (lane 4) for 24 hours. As shown, FasL+CHX could induce cleavage of the inactive form of caspase 3 and this effect was partially inhibited by co-incubation with IGF-I. The barogram summarises the results obtained in two independent experiments: * *P *< 0.05 when compared with incubation with FasL+CHX without IGF-I.

Finally, the anti-apoptotic effects of IGF-I were further evaluated on other effector mechanisms, that is, the cleavage of PARP and caspase 3 (two enzymes involved in the final steps of apoptosis). As shown in Figure 6, exposure of human HSCs to FasL+CHX induces cleavage of PARP and this effect is partially reversed by co-incubation with IGF-I. In addition, the cleavage of caspase 3 induced by FasL+CHX was decreased by co-incubation with IGF-I (Figure 7).

## Discussion

The reversibility of fibrosis and even cirrhosis is currently a central issue in hepatology. The introduction of more effective anti-viral treatments and possibly anti-fibrogenic agents is directed at reducing fibrosis as a key end-point [29]. In this context, a clear definition of the cellular and molecular mechanisms regulating apoptosis of fibrogenic cell types, including HSCs, is urgently required. In addition, affinities and differences between experimental models and human disease need to be better defined and clarified. It is evident that in experimentally induced liver fibrosis in rodents, cessation of liver injury results in fibrosis regression, usually associated with reduction of TIMP-1 expression and HSC apoptosis. These observations are supported by in vitro studies performed in activated rodent HSCs [7-10]. Based on this evidence, clearance of activated HSCs by apoptosis has been regarded as an appealing target for anti-fibrotic therapy [30]. However, the regulation of apoptosis in activated human HSCs deserves further evaluation. Novo et al. [13] have demonstrated that activated human HSCs do not undergo spontaneous apoptosis and survive when exposed to prolonged serum deprivation and numerous other pro-apoptotic stimuli [31]. Induction of caspase-dependent, mitochondria-driven apoptosis in human HSCs was observed only when actinomycin D or cycloheximide were added to the culture, indicating that de novo protein expression contributes to resistance to apoptotic stimuli. In particular, these authors observed an increasingly higher expression of BCl-2 during the process of HSC activation. The possibility that human HSCs respond to pro-apoptotic stimuli differently from rodent cells has raised the need for a more extensive characterisation of the responsible mechanisms and pathways involved in this process. Accordingly, the aim of the present study was to investigate the involvement of other key anti-apoptotic pathways such as PI-3K/Akt/p-Bad in response to IGF-I. The choice of IGF-I as a stimulus for these investigations was based on extensive evidence of this polypeptide as a potent survival factor. It has been shown in numerous cell types that IGF-I acts through the activation of PI-3K and several downstream molecules. In addition, other pathways are likely to be implicated in the cell survival action of IGF-I, particularly ERK-kinase activation, Raf activation and p38 activation [32].

The results of the present study confirmed that in activated human HSCs, IGF-I induced the activation of molecules downstream of PI-3K. In particular, it was observed that IGF-I can induce Akt activation and phosphorylation of Ser 473 located in the C-terminal regulator domain of the protein and this effect is totally dependent on PI-3K activation since it was completed inhibited by wortmannin or LY294002. Phosphorylation at this site results in the binding of Bad to 14-3-3t protein, thus inhibiting Bad binding to Bcl-2 and Bcl-Xl. Of note, IGF-I-induced Bad phosphorylation was not completely reversed by PI-3-K inhibitors. This could be due to the fact that other IGF-I activated proteins able to phosphorylate Bad are not activated by PI-3K. In this context we could exclude the involvement of either ERK or PKA activation in Bad phosphorylation (data not shown).

In addition, exposure to IGF-I for 24 hours induced an increased expression of the anti-apoptotic protein Bcl-Xl, an anti-apoptotic protein that binds Bad. Taken together, these data indicate that IGF-I could protect cells from apoptosis acting both on anti-apoptotic signalling and the expression of anti-apoptotic proteins.

We then evaluated the involvement of GSK3β in IGF-I-induced PI-3K activation. GSK3β was initially identified as an enzyme that regulates glycogen synthesis in response to insulin. GSK3β is a ubiquitously expressed serine/threonine protein kinase that phosphorylates and inactivates glycogen synthase. GSK3β has been shown to regulate cyclin D1 proteolysis and subcellular localisation [33]. GSK3β knock-out mice show accelerated wound closure and fibrogenesis, thus suggesting an inhibitory role of this kinase [34]. In our experimental setting, IGF-I induced the phosphorylation of GSK3β after 15 minutes of incubation, and this effect was PI-3K-dependent. This observation provides additional molecular insights into the pro-survival action of IGF-I and reinforces its role in the fibrogenic process.

Other downstream targets of Akt are the FOXO family of transcription factors. Phosphorylation of FKHR family members by Akt promotes cell survival and regulates the cell cycle. Phosphorylation of FKHR protein regulates their nuclear translocation and target gene transcription [35]. Our data indicate that IGF-I induces the phosphorylation of Fox 1 and Fox 4 of the Forkhead family and this phosphorylation is strongly reduced by pre-incubation with WMN, thus confirming a predominant anti-apoptotic action of this growth factor through the activation of PI-3K and related downstream pathways.

Finally, a dedicated set of experiments confirmed the apoptosis-resistant phenotype of this activated human HSC. Numerous factors were used to induce human HSC apoptosis but only with high doses of FasL+cyclohexymide, were caspase 3 and PARP cleavage observed. In support of the survival action of IGF-I, incubation with this growth factor resulted in a partial reversion of this effect.

## Conclusion

In conclusion, the results of the present study provided additional insight into the regulation of apoptosis of human HSCs, a key cell type involved in hepatic fibrogenic disorders. Human HSCs in their MF-like phenotype are characterised by the activation of several anti-apoptotic pathways. This leads to a constitutive apoptotic-resistant phenotype that is further supported by the presence of potent survival factors such as IGF-I. These features likely contribute to the limited reversibility of long-term liver fibrosis when the cause of damage is successfully removed. Accordingly, the information provided by this study will be instrumental in designing pharmacological strategies able to promote HSC apoptosis.

## Abbreviations

ECL: enhanced chemiluminescence; EDTA: ethylenediaminetetraacetic acid; EGTA: ethylene glycol tetraacetic acid; FOXO: Forkhead box O; GSK3β: glycogen synthase kinase 3β; HEPES: 4-(2-hydroxyethyl)-1-piperazineethanesulphonic acid; HSC: hepatic stellate cell; IGF-I: insulin-like growth factor I; MF: myofibroblast; NGF: nerve growth factor; PARP: poly (ADP-ribose) polymerase; PDGF: platelet-derived growth factor; SDS-PAGE: sodium dodecyl sulphate polyacrylamide gel electrophoresis; TIMP: tissue inhibitor of metalloproteinase; TNF: tumour necrosis factor.

## Competing interests

The authors declare that they have no competing interests.

## Authors' contributions

AG performed most of the experiments and wrote the papers. MP supervised the work and wrote the paper. FM wrote part of the paper. AC performed experiments on AMP cyclic production induced by IGF-I. BL performed with MB some western blots, LC performed experiments on HSC apoptosis.
